# Comparison of temporal changes in psychological distress after hematopoietic stem cell transplantation among the underlying diseases of Japanese adult patients

**DOI:** 10.1186/1751-0759-2-24

**Published:** 2008-11-21

**Authors:** Wataru Fukuo, Kazuhiro Yoshiuchi, Yoshiyuki Takimoto, Noriyuki Sakamoto, Hiroe Kikuchi, Maki Hachizuka, Shuji Inada, Yasuhito Nannya, Keiki Kumano, Tsuyoshi Takahashi, Mineo Kurokawa, Akira Akabayashi

**Affiliations:** 1 Department of Stress Sciences and Psychosomatic Medicine, Graduate School of Medicine, the University of Tokyo, Tokyo, Japan; 2 Department of Hematology/Oncology, the University of Tokyo, Tokyo, Japan; 3 Department of Cell Therapy and Transplantation Medicine, the University of Tokyo, Tokyo, Japan

## Abstract

**Background:**

Although hematopoietic stem cell transplantation (HSCT) can potentially cure some hematological malignancies, patients who undergo HSCT experience psychological distress. However, there have been few studies on the short-term influence of HSCT on psychological distress.

**Methods:**

The subjects were 71 patients with hematological malignancies who underwent HSCT: 33 with acute leukemia, 19 with chronic leukemia, nine with myelodysplastic syndrome, and 10 with malignant lymphoma. Psychological distress was assessed prior to HSCT and on the seventh day after HSCT using the Profile of Mood States (POMS).

**Results:**

With regard to Anger-Hostility, the interaction of time (pre- and post-HSCT) and group (the four groups) was significant in male patients (p = 0.04), but not in female patients. With regard to the other subscales of POMS, there was no significant main effect or interaction in male or female patients.

**Conclusion:**

It may be important to provide psychological support to patients throughout the period of HSCT in consideration of differences in mood changes associated with the underlying disease and patient sex in order to provide efficient psychiatric intervention for both better psychiatric and survival outcomes.

## Findings

Hematopoietic stem cell transplantation (HSCT) is an alternative to conventional treatment for patients with hematological malignancies and can potentially cure several malignant diseases. However, about one-third to two-thirds of patients treated with allogeneic HSCT die due to a relapse of the disease or from procedure-related complications such as organ damage and graft-versus-host disease (GVHD) [1,2]. As such, HSCT is associated with life-threatening physical morbidity. In addition, patients undergoing HSCT are obligated to stay in a germ-free ward for several weeks where they suffer from social isolation. They also have to wait at least two or three weeks until the success of the HSCT procedure becomes evident, which can influence their psychological state [3].

It has previously been reported that psychological distress after allogeneic HSCT may vary with the underlying disease due to differences in chemotherapies prior to HSCT[4,5]. In addition, although there have been some studies on the psychosocial impact of HSCT on patients undergoing allogeneic HSCT, most of them investigated comparatively long-term influence [6,7] although Hjermstad et al. [8] reported the course of anxiety and depression in HSCT patients from two weeks to one year after HSCT.

Therefore, the aim of this study was to compare the short-term changes of psychological distress induced by allogeneic HSCT before the advent of successful engraftment in adult Japanese patients with various underlying diseases.

Subjects were patients with hematological malignancies who underwent HSCT at The University of Tokyo Hospital. The inclusion criteria were as follows: a) at least 18 years of age; b) a diagnosis of either acute or chronic leukemia, myelodysplastic syndrome (MDS), or malignant lymphoma; and c) received allogeneic HSCT between September 1996 and April 2006 at The University of Tokyo Hospital. Patients were asked to complete the Profile of Mood States (POMS) [9] twice – once before entering the germ-free ward and a second time on the seventh day after HSCT. After HSCT, the waiting period for successful engraftment is at least two or three weeks. Therefore, we chose the 7th day as the post-HSCT assessment point to investigate mood states at a time well before the success of the engraftment became evident. POMS consisted of the following six subscales: Tension-Anxiety, Depression, Anger-Hostility, Vigor, Fatigue, and Confusion. The mean scores (SD) of Tension-Anxiety, Depression, Anger-Hostility, Vigor, Fatigue, and Confusion for men in the Japanese general population (n = 3154) are 12.0 (6.3), 9.9 (9.8), 10.8 (8.2), 14.2 (6.1), 9.3 (6.2), 8.6 (4.7), respectively while those for women in the Japanese general population (n = 2423) are 12.1 (7.1), 10.9 (10.6), 10.9 (8.8), 13.3 (6.2), 10.2 (6.6), 8.7 (4.8), respectively [9].

Repeated measures analysis of variance (ANOVA) was used to compare temporal changes of each subscale of POMS among four groups: Acute leukemia, chronic leukemia, myelodysplastic syndrome (MDS), and malignant lymphoma). We separately analyzed male and female patients because Andorykowski et al. [6] reported that the influence of HSCT on psychological status was different between men and women. Age was also compared among the four groups using ANOVA.

All the procedures and materials were approved by the institutional review board of the University of Tokyo and informed consent was obtained from all subjects.

Seventy-one out of 200 eligible patients completed POMS twice. Male patients consisted of 22 with acute leukemia (of 64 eligible), 11 with chronic leukemia (of 24 eligible), six with MDS (of 22 eligible), and six malignant lymphoma patients (of 14 eligible). Female patients consisted of 11 with acute leukemia (of 38 eligible), eight with chronic leukemia (of 18 eligible), three with MDS (of five eligible) and four lymphoma patients (of 15 eligible). There was no significant difference in age among the four male groups (mean ± SD years: acute leukemia, 37.0 ± 11.9; chronic leukemia, 35.6 ± 10.4; MDS, 45.2 ± 10.0; malignant lymphoma, 39.7 ± 13.8) or in the female group (mean ± SD years: acute leukemia, 29.6 ± 10.4; chronic leukemia, 42.8 ± 12.6; MDS, 31.0 ± 7.0; malignant lymphoma, 35.5 ± 16.1).

**Figure 1:**
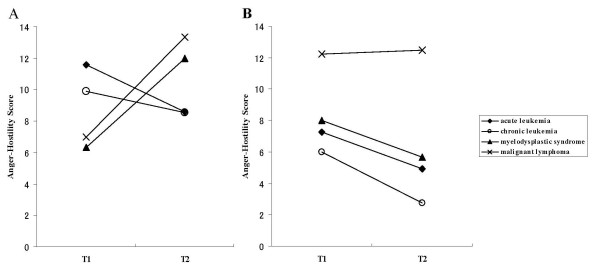
**Temporal changes in POMS Anger-Hostility score before and after HSCT**. Changes in Anger-Hostility scores between pre- and post-hematopoietic stem cell transplantation (HSCT) are displayed by disease group. For male patients, the interaction of time and group was significant (p = 0.04) (A). However, for female patients there was no significant main effect or interaction (B). Patients with higher Anger-Hostility scores have more distress. T1, time point of pre-HSCT; T2, seventh day after HSCT. The mean score (SD) of Anger-Hostility for men in the Japanese general population (n = 3154) is 10.8 (8.2) while that of women in the Japanese general population (n = 2423) is 10.9 (8.8) [9].

With regard to Anger-Hostility, the time × group interaction was significant in male patients (p = 0.04) although mean scores were lower than the mean + SD score for Anger-Hostility for men in the Japanese general population, while there was no significant effect for female patients (Figure 1). As shown in Figure 1, Anger-Hostility scores in the male MDS and malignant lymphoma groups worsened, whereas there was no change in the other two groups. With regard to the other subscales of POMS, there was no significant main effect or interaction for male or female patients.

Contrary to the previous study [4], Anger-Hostility scores in the chronic leukemia group did not worsen compared to the other groups. The reason for this discrepancy might be that many chronic leukemia patients in the present study had undergone intensive chemotherapy before HSCT due to blastic crisis or acute exacerbation.

In the present study, the Anger-Hostility scores of patients with MDS, who had not undergone intensive chemotherapy, worsened after HSCT. Examples of Anger-Hostility might include unfocused anger such as "Why is this happening to me?" This result is consistent with a previously reported study [4] where patients with chronic leukemia felt more distress than those with acute leukemia because many of the patients with chronic leukemia had not gone through intensive chemotherapy. Future studies are needed to investigate the reason for the temporal pattern of change in Anger-Hostility scores in patients with malignant lymphoma.

In the present study, the time × group interaction in Anger-Hostility was significant **for **male patients but not for female patients. There have been few studies on the influence of gender on psychological states after HSCT. One study by Sasaki et al. [10] reported that being female was one of the risk factors for developing mental disturbances during isolation. The reason for this difference in results concerning gender effects on psychological states after HSCT compared to the previous study is not clear because the time of the assessment was not the same in the two studies. Therefore, further prospective studies are needed to investigate the influence of gender on the psychological state after HSCT.

In the present study we observed a significant interaction of time (pre- and post-HSCT) and group (the four groups) in Anger-Hostility (p = 0.04) but not in Tension-Anxiety or in Depression. These results may partly be due to the time of the second assessment (one week after HSCT) of psychological distress. Hjermstad et al. [8] reported that depressive mood peaked two to four weeks after HSCT and that anxiety gradually decreased four weeks after HSCT. However, further studies are needed to investigate prospective changes in anger or hostility because there has been no study on anger or hostility after HSCT.

There are some limitations in the present study. First, the sample sizes of some groups were small, especially for female patients, thus we could not detect differences among the groups. Second, many potential subjects who were eligible did not participate. Finally, some factors that might influence psychosocial variables, such as family function [11,12], the presence of a spouse [11] and physical functioning [13,14] were not controlled or investigated.

In conclusion, it may be important to support patients throughout the period of HSCT with consideration to differences in mood changes that are associated with the underlying disease and patient sex. Because there have been studies that address mental disorders during HSCT [10] and the effect on transplant outcomes of early psychological distress [8], further studies on the influence of factors such as gender, psychosocial factors and chemotherapy before HSCT on mood changes during HSCT may contribute to efficient psychiatric intervention for both better psychiatric and survival outcome.

## Competing interests

The authors declare that they have no competing interests.

## Authors' contributions

WF designed the study, collected the data, performed statistical analyses, interpreted the results, and drafted the manuscript. KY designed the study, collected the data, performed statistical analyses, interpreted the results, and drafted the manuscript. YT interpreted the results and drafted the manuscript. NS, HK, MH, and SI collected the data, interpreted the results, and drafted the manuscript. YN, KK, and TT helped collect the data, interpret the results, and draft the manuscript. MK and AA helped interpret the results, and draft the manuscript. All authors read and approved the final manuscript.
